# Retraction: Valproic Acid Attenuates Traumatic Brain Injury-Induced Inflammation *in Vivo*: Involvement of Autophagy and the Nrf2/ARE Signaling Pathway

**DOI:** 10.3389/fnmol.2026.2038844

**Published:** 2026-10-28

**Authors:** Frontiers Editorial Office

The journal retracts the 17 April 2018 article cited above.

Following publication, concerns were raised regarding the integrity of the images in the published Figures 3, 4, 5 and 6. The same images were used in different studies to represent different experimental conditions, and in two instances panels appear to overlap with rotation within the same figure. An investigation was conducted in accordance with Frontiers' policies. While the authors provided raw data and images, following an assessment of these and due to the severity of the issues, the conclusions of the article have been deemed unreliable, and the article has been retracted.

This retraction was approved by the Chief Editors of Frontiers in Molecular Neuroscience and the Chief Executive Editor of Frontiers. The authors received a communication regarding the retraction and had a chance to respond. This communication has been recorded by the publisher.

Frontiers would like to thank the users on PubPeer for bringing the published article to our attention.

